# A Comprehensive Analysis of 2013 Dystrophinopathies in China: A Report From National Rare Disease Center

**DOI:** 10.3389/fneur.2020.572006

**Published:** 2020-09-30

**Authors:** Yuan-Ren Tong, Chang Geng, Yu-Zhou Guan, Yan-Huan Zhao, Hai-Tao Ren, Feng-Xia Yao, Chao Ling, Dan-Chen Wang, Lin Chen, Li-Ying Cui, Shu-Yang Zhang, Yi Dai

**Affiliations:** ^1^Peking Union Medical College Hospital, Chinese Academy of Medical Sciences & Peking Union Medical College, Beijing, China; ^2^Department of Neurology, Peking Union Medical College Hospital, Chinese Academy of Medical Sciences & Peking Union Medical College, Beijing, China; ^3^Laboratory of Clinical Genetics, Peking Union Medical College Hospital, Chinese Academy of Medical Sciences & Peking Union Medical College, Beijing, China; ^4^Department of Clinical Laboratory, Peking Union Medical College Hospital, Chinese Academy of Medical Sciences & Peking Union Medical College, Beijing, China; ^5^Department of Cardiology, Peking Union Medical College Hospital, Chinese Academy of Medical Sciences & Peking Union Medical College, Beijing, China

**Keywords:** NRDRS, Duchenne muscular dystrophy, Becker muscular dystrophy, hospital-registry, mutation spectrum, rare mutations

## Abstract

Duchenne muscular dystrophy (DMD) and Becker muscular dystrophy (BMD) are X-linked recessive neuromuscular disorders caused by mutations in *DMD*. A high-quality database of DMD/BMD is essential not only for clinical practice but also for fundamental research. Here, we aimed to build the largest Chinese national dystrophinopathy database using the National Rare Diseases Registry System of China. Peking Union Medical College Hospital (PUMCH) was the National Rare Diseases Center of China. This research involved 2013 patients with dystrophinopathies, whose diagnoses were confirmed; they were registered and followed up at PUMCH from March 2011 to December 2018. Family history, clinical signs, and treatment data were reported for patients with DMD and BMD at different rates. All six serum biochemical indexes could accurately distinguish between DMD and BMD patients. Copy number variations were the most frequent mutation type (79.2% in DMD and 84.3% in BMD), of which large deletions accounted for 88.4 and 88.6%, large duplications accounted for 11.6 and 11.4% in DMD and BMD, respectively. An exon deletion hotspot, located in exons 45–54, was observed in DMD, and intron 44 was the most frequent deletion starting point (26.5%). Duplication and single nucleotide variations appeared to be uniformly distributed among all exons. Eleven patients were identified to have ultrarare mutation types. Eleven other patients suffered from two separate mutations simultaneously, some of which may have taken place via dependent mechanisms. Thus, we have established the largest hospital-based Chinese dystrophinopathy database via the National Rare Diseases Registry System. This study provides valuable information for further diagnostic and therapeutic studies of dystrophinopathy.

## Introduction

Duchenne muscular dystrophy (DMD), Becker muscular dystrophy (BMD), and intermediate muscular disease (IMD) are X-linked recessive neuromuscular disorders caused by mutations in *DMD* (MIM: 310200, ORPHA: 98896) (1, 2). The incidence of DMD is 1/4,709 in China and ranges from 1/3,802 to 1/6,291 in male births according to newborn screening studies across different countries (3, 4). The prevalence of BMD ranges from 1/4 to 1/3 of that of DMD according to population-based studies (5–7), yet the incidence of BMD may be underestimated, since subclinical BMD might have escaped epidemiological survey (8). IMD were accompanied with heterogeneous clinical manifestations, whose severity was between DMD and BMD.

Affected DMD patients are usually diagnosed because of abnormal gait from 2 to 5 years old, and those who do not receive medical treatment lose the ability to walk before they are 12 years old (9). Patients with BMD usually have a relatively later onset and less severe symptoms, including slow-progressing muscle weakness yet more frequent cardiomyopathy (10, 11). In most cases, ambulation remains until the patient is older than 16 years. Some even do not present any symptoms until they are 30 years old (12).

Biomarkers, especially significantly elevated creatine kinase (CK) and lactate dehydrogenase (LDH), released from damaged muscle cells, contribute to clinical diagnosis (13–15). However, abnormal biomarker levels might also be caused by other diseases, such as hepatitis. Genetic testing for *DMD* is recommended as the first-line test to confirm a diagnosis (16). Results of genetic testing are also essential for patients who expect a novel therapy, including non-sense read-through therapy, exon-skipping therapy, and genetic therapy (17).

*DMD* is composed of 79 exons, spanning over 2.5 million nucleotides. Mutations on *DMD* can be divided into copy number variations (CNVs) and single nucleotide variations (SNVs). CNVs can be further divided into deletion or duplication of one or more exons, while SNVs include non-sense, missense, splice-site mutations, and small insertions or deletions. Deletion of one or more exons makes up the majority of the mutations, ranging from 43 to 80% (18–20). A high-quality database of DMD/BMD is of vital importance, not only for clinical practice but also for fundamental medical research, especially for new therapies based on genetic patterns (17). However, until now, only a few medical centers in China have collected sufficient DMD/BMD patient information with unified criteria. Previous Chinese DMD databases either contain a small sample size (21, 22) or are based on an internet-registry of self-reported information (23).

Here, we built a database supported by the National Rare Diseases Registry System of China (NRDRS-DMD/BMD database), not only increasing the number of involved patients but also following a standardized hospital-based registry. This article reports the genetic variations, along with demographics, clinical features, biomarkers, and treatment data based on the database. We hope to lay the foundation for further research, exploring potential pathophysiological mechanisms and novel therapies for patients with dystrophinopathy.

## Materials and Methods

All patients involved in the study provided informed consent before involvement, and this study was approved by the ethics committee of the Peking Union Medical College Hospital (IRB #JS-1233).

### Patients

Consecutive outpatients were referred to the Peking Union Medical College Hospital (PUMCH) neuromuscular center by local hospitals, as PUMCH was the referral hospital for inherited neuromuscular diseases and the National Rare Disease Center in China. The NRDRS-DMD/BMD database was built in 2011 and cooperated with the Translational Research in Europe-Assessment and Treatment of Neuromuscular Diseases (TREAT-NMD). Some items included in the database were also included in the TREAT-NMD registry (24).

According to standard guidelines (16), genetic diagnosis, immunohistochemistry, immunofluorescence and western blotting could be used to confirm NMD and genetic diagnosis was the first recommendation. Result of genetic diagnosis was used as major inclusion criteria in this research.

Patients were evaluated by the neuromuscular specialists and classified into DMD, BMD, and IMD subgroups according to clinical profiles, physical examinations, laboratory tests, and genetic results, which complied with the standard guidelines (16). Comprehensive characteristics were collected from the database.

Patients with CNVs were detected using a multiplex ligation-dependent probe amplification (MLPA) kit (MRC-Holland, The Netherlands). Patients with SNVs were detected using whole *DMD* capture and sequencing (NGS + MygenoCap; MyGenotics), as explained previously (25). Finally, patients without any positive genetic results were recommended for muscle biopsy and RNA sequencing of the muscle tissue. In this study, we analyzed the registry data from March 2011 to November 2018.

### Muscle Biopsy and RNA Sequencing

Muscle biopsy and pathological analysis were performed for patients without mutations, detected using MLPA and sequencing. In addition to regular staining, immunohistochemical staining of dystrophin N-terminus (NCL -DYS3), C-terminus (NCL-DYS2), and rod (NCL-DYS1), using mouse monoclonal antibodies from the Novocastra lab, was performed to measure the expression of dystrophin in the muscle fiber membrane, following standard protocols (26).

The mRNA library was prepared from fresh muscle tissues obtained via muscle biopsy, as described previously (27). cDNA was generated using random primers (27) and then amplified as 10–20 overlapping fragments (28). After quality control, Illumina HiSeq X platform was used for sequencing the amplified products in 150 bp paired-end reactions. Downstream data analysis consisted of quality control, sequencing adapter trimming, and removal of reads with poor quality scores followed by mapping reads. Deep intronic mutations were identified and analyzed after RNA sequencing.

### Statistical Analysis

Receiver operating characteristic curves (ROC) were used to calculate the cutoff values of CK, LDH, creatine kinase-muscle/brain (CK-MB), hydroxybutyric dehydrogenase (HBD), aspartate aminotransferase (AST), and alanine aminotransferase (ALT) levels between patients with DMD and BMD. Student's *t-*tests were performed to compare indexes between patients with DMD and BMD. The threshold of statistical significance was set at 0.05. All statistical analyses were performed using R software 3.5.1.

## Results

### Demographic Analysis

A total of 2,013 patients were enrolled in our registry from March 2011 to December 2018, among whom 2,013 patients were males, and one patient was female (which was clarified as rare pathophysiology). A total of 1,972 patients were diagnosed based on genetic results (by MLPA and/or sequencing), 11 patients were diagnosed based on muscle biopsy, and 30 patients had an incomplete genetic diagnosis but refused to undergo additional tests (Supplementary Figure 1). Among the clinical types, there were 1,544 DMDs, 368 BMDs, and 101 IMDs.

**Figure 1:**
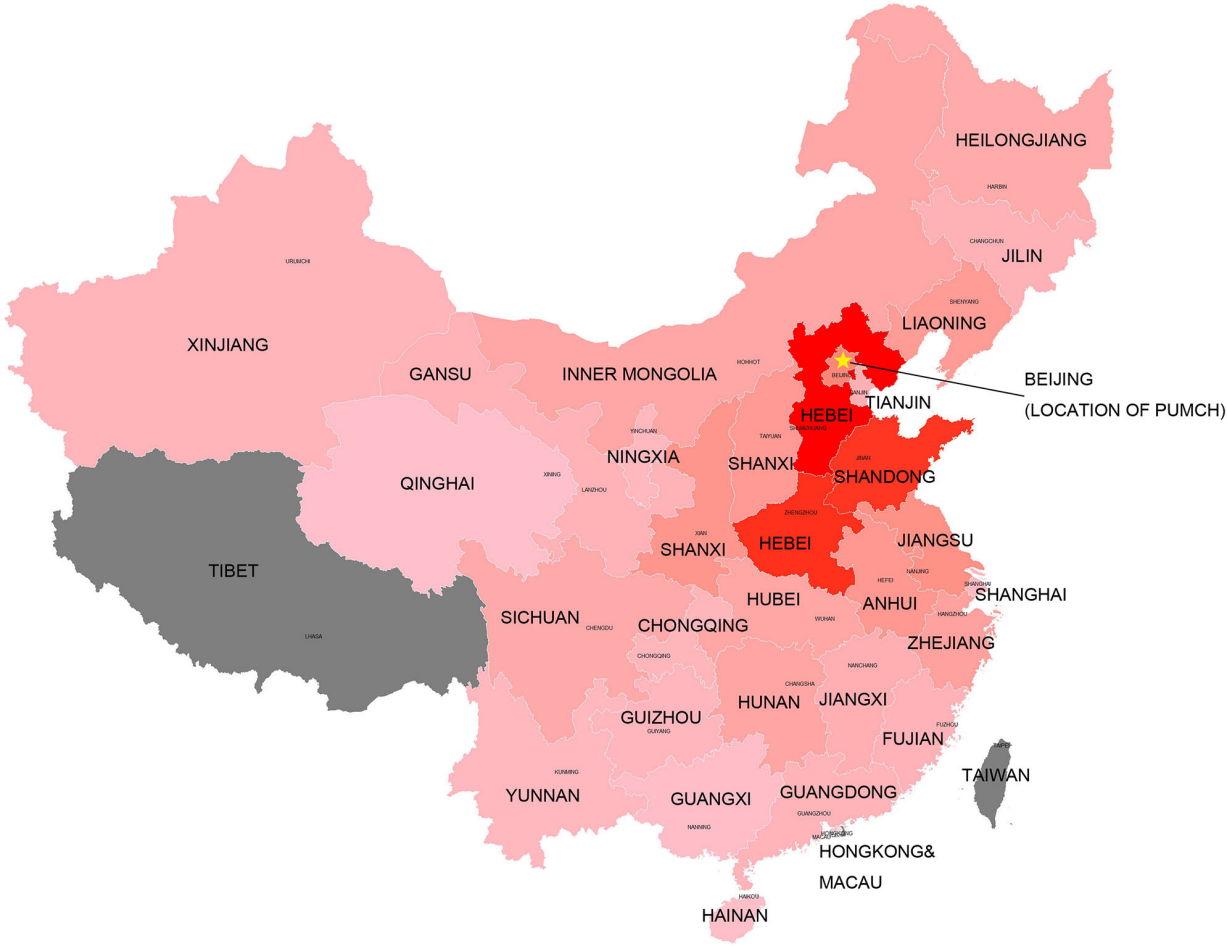
Registered patients in each province of China. The “star” indicates the location of the medical center (PUMCH). The dark red color indicates larger number of registered patients in this province. The gray color indicates no sufficient data included in the study.

Patients resided mostly in regions of China, as well as Tibet, Macau, and Taiwan (Figure 1). The top five regions were Hebei (*n* = 280, 13.9%), Henan (*n* = 255, 12.6%), Shandong (*n* = 247, 12.3%), Beijing (*n* = 120, 6.0%), and Jiangsu (*n* = 98, 4.9%). Beijing (6.12), Hebei (3.91), Tianjin (2.78), Henan (2.71), and Shandong (2.58) had the highest enrolled patient rates (1 patient per million residents) when the resident population of each region was taken into consideration. Patient numbers and registration rates in each province are shown in Supplementary Table 1. Figure 1 indicates that the number of registered patients in each province was inversely proportional to the distance of the province to the capital-Beijing, where PUMCH is located.

### Clinical Information

Demographics, clinical information, biomarkers, and treatment data are summarized in Table 1. The median age of included patients was 9 (range: 1–32) for DMD, 9 (range: 1–67) for BMD, and 10 (range: 2–34) for IMD, while the median diagnosis age was 4 for DMD (range: 0–16), 4 for BMD (range: 0–41), and 6 for IMD (range: 0–30). Patients with DMD were related to a significantly higher concentration of all six serum enzymes than those with BMD in our database. Specificity, sensitivity, and area under the curve (AUC) were calculated. Based on the results, all six biochemical indexes could distinguish DMD and BMD patients correctly (Supplementary Table 2). Treatment data from patients with DMD were also included in the database. Precisely 74.8% of patients currently and previously used steroids and only 1.5% of patients received different types of ventilation measures.

**Table 1:** Demographics, clinical features, biomarker levels, and treatment data of patients.

**Features**		**BMD *N =* 368**	**DMD *N =* 1,544**	**IMD *N =* 101**
**Age distribution**				
Age at registry, median (range)^*^		9 (1–67)	9 (1–32)	10 (2–34)
Age at diagnosis, median (range)		4 (1–41)	4 (0–16)	6 (0–30)
**Clinical features**				
Family history, n (%)	No	170 (46.2%)	1,020 (66.0%)	62 (61.4%)
	Yes	62 (16.8%)	131 (8.5%)	15 (14.9%)
	Unknown	136 (37.0%)	393 (25.5%)	24 (23.7%)
Ability of crawling, n (%)	No	52 (14.1%)	616 (39.9%)	29 (28.7%)
	Poor	36 (9.8%)	215 (13.9%)	26 (25.8%)
	Regular	30 (8.2%)	15 (1.0%)	6 (5.9%)
	Unknown	250 (67.9%)	698 (45.2%)	40 (39.6%)
Ability of running, n (%)	No	0 (0.0%)	27 (1.8%)	2 (2.0%)
	Slow	93 (25.3%)	1,043 (67.5%)	68 (67.3%)
	Regular	39 (10.6%)	5 (0.3%)	2 (2.0%)
	Unknown	236 (64.1%)	469 (30.4%)	29 (28.7%)
Gowers' sign, n (%)	+	27 (7.3%)	378 (24.5%)	26 (25.7%)
	±	14 (3.8%)	172 (11.1%)	39 (38.6%)
	–	128 (34.8%)	112 (7.2%)	33 (32.7%)
	Unknown	199 (54.1%)	882 (57.2%)	3 (3.0%)
Calf pseudohypertrophy, n (%)	+	202 (54.9%)	1,033 (66.9%)	70 (69.3%)
	±	32 (8.7%)	106 (6.9%)	1 (1.0%)
	–	5 (1.4%)	11 (0.7%)	6 (5.9%)
	Unknown	129 (35.0%)	394 (25.5%)	24 (23.8%)
Forearm pseudohypertrophy, n (%)	+	125 (34.0%)	588 (38.1%)	39 (38.6%)
	±	57 (15.5%)	297 (19.2%)	21 (20.8%)
	–	47 (12.8%)	218 (14.1%)	10 (9.9%)
	Unknown	139 (37.2%)	441 (28.6%)	31 (30.7%)
**Biomarkers**				
CK, mean ± SD		5412.4 ± 5998.5	17293.8 ± 10516.0	15222.7 ± 11191.6
CK-MB, mean ± SD		114.6 ± 95.2	466.8 ± 457.5	367.5 ± 453.3
LDH, mean ± SD		578.2 ± 278.6	1410.6 ± 1088.6	1331.0 ± 2672.7
AST, mean ± SD		121.9 ± 67.4	290.5 ± 156.9	252.0 ± 257.1
ALT, mean ± SD		138.0 ± 90.1	368.0 ± 146.0	290.0 ± 140.2
HBD, mean ± SD		484.5 ± 241.5	999.7 ± 351.1	788.7 ± 292.3
**Treatment**^**#**^				
**Steroid, n (%)**^***$***^				
Currently receiving steroids		/	544 (35.2%)	/
Previously receiving steroids		/	611 (39.6%)	/
Never receiving steroids		/	164 (10.6%)	/
Unknown		/	225 (14.6%)	/
**Ventilation, n (%)**	
No ventilation		/	1,304 (84.4%)	/
Part-time non-invasive ventilation		/	15 (1.0%)	/
Full-time ventilation		/	6 (0.4%)	/
Other ventilation		/	2 (0.1%)	/
Unknown		/	218 (14.2%)	/

* *This study involved patients registered before 2018.12.31*.

# *Treatment data were only included in DMD patients*.

$ *Patients only with short-term steroid use were also included*.

*CK, creatine kinase; CK-MB, creatine kinase-muscle/brain; LDH, lactate dehydrogenase; AST, aspartate aminotransferase; ALT, alanine aminotransferase; HBD, hydroxybutyric dehydrogenase*.

The patients involved were divided into different age groups, as shown in Supplementary Table 3, presenting with different distributions of clinical features and treatment data. The 5–14 year group in DMD patients presented with the highest ratio of steroid treatment (80.9%, including both currently and previously receiving steroids) and 65.2% for patients younger than 5 years.

### Mutation Types and Frequencies

A total of 1,972 patients had confirmed genetic test results, including CNVs, SNVs, and ultrarare mutations. CNVs were the most frequent mutation type (79.2, 84.3, and 80.6%, respectively), among which large deletions accounted for 88.4, 88.6, and 72.2%, and large duplications accounted for 11.6, 11.4, and 27.9% in patients with DMD, BMD, and IMD, respectively. For DMD, SNVs accounted for 19.7%, among which non-sense mutations accounted for 52.7%, followed by small deletions (22.5%), splice mutations (16.1%), small insertions (6.7%), and missense mutations (2.0%). For BMD, SNVs accounted for 14.3%, among which splice mutations accounted for 38.5%, followed by non-sense mutation (25.0%), small deletions (15.4%), missense mutations (15.4%), and small insertions (5.8%). For IMD, SNVs accounted for 18.4%, among which non-sense mutations and small deletions accounted for 27.8%, followed by splice site mutations (22.2%), missense mutations (11.1%), and small insertions (11.1%).

### CNVs

**Figure 2:**
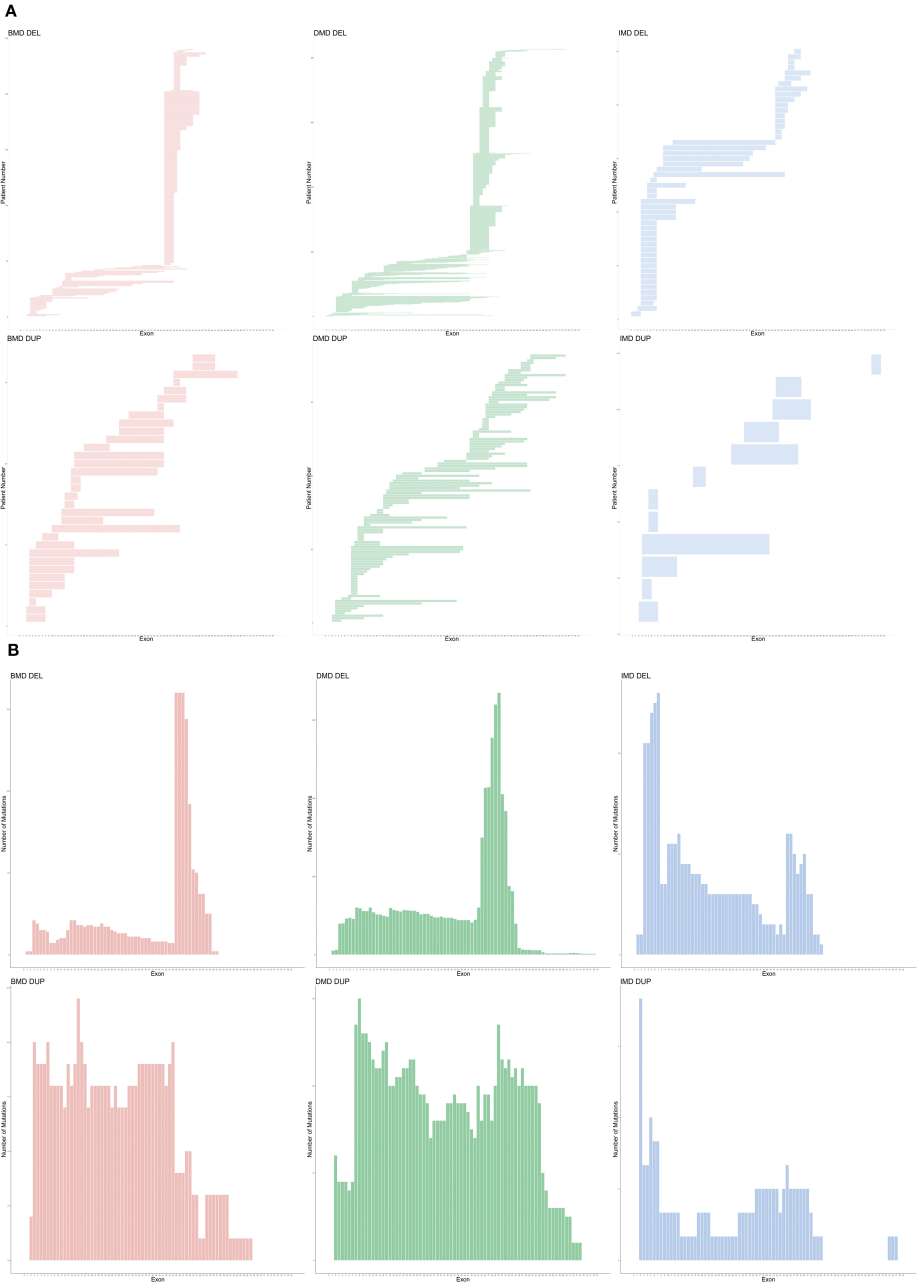
Genetic pattern of patients with CNVs. **(A)** Mutation pattern of individual patients. Each horizontal line shows the range of deletion or duplication of one patient. **(B)** Cumulative distribution of CNV in all exons. CNV, copy number variations; DEL, deletion; DUP, duplication.

**Figure 3:**
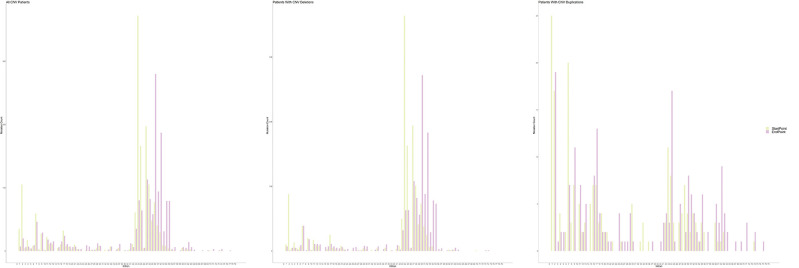
Distribution of starting and ending breakpoints of CNV mutations. Each column represents with the cumulative number of starting/ending breakpoints at each introns. CNV, copy number variation.

An exon deletion hotspot located in exons 45–54 was observed in DMD, which accounted for 72.1% of all DMD deletions, as shown in Figure 2. For BMD, deletion for exons 45–55 accounted for 77.9% of all BMD deletions. A total of 187 and 56 deletion types existed in DMD and BMD, respectively. Deletion of exons 48–50 was the most frequent type (7.5%) in DMD, and deletion of exons 45–47 was the most common type in BMD (22.9%). Deletion after exon 56 was rare in our database, accounting for only 1.5 and 0.7% of DMD and BMD cases, respectively. Intron 44 was the most frequent deletion starting point (26.5%) in all patients, while intron 50 and 52 accounted for the top 2 frequent (19.6 and 13.2%) at the endpoints, as presented in Figure 3.

For duplication, no apparent hotspot region existed in either DMD or BMD in our database. A total of 97 and 28 duplication types in DMD and BMD was observed, respectively, while each duplication type had a similar proportion.

For IMD, a different pattern was observed; exon 3–7 deletions was the most frequent (26.3%) among all deletions, while exon 2 duplication accounted for the most frequent (4.1%) among all duplications.

### SNVs

**Figure 4:**
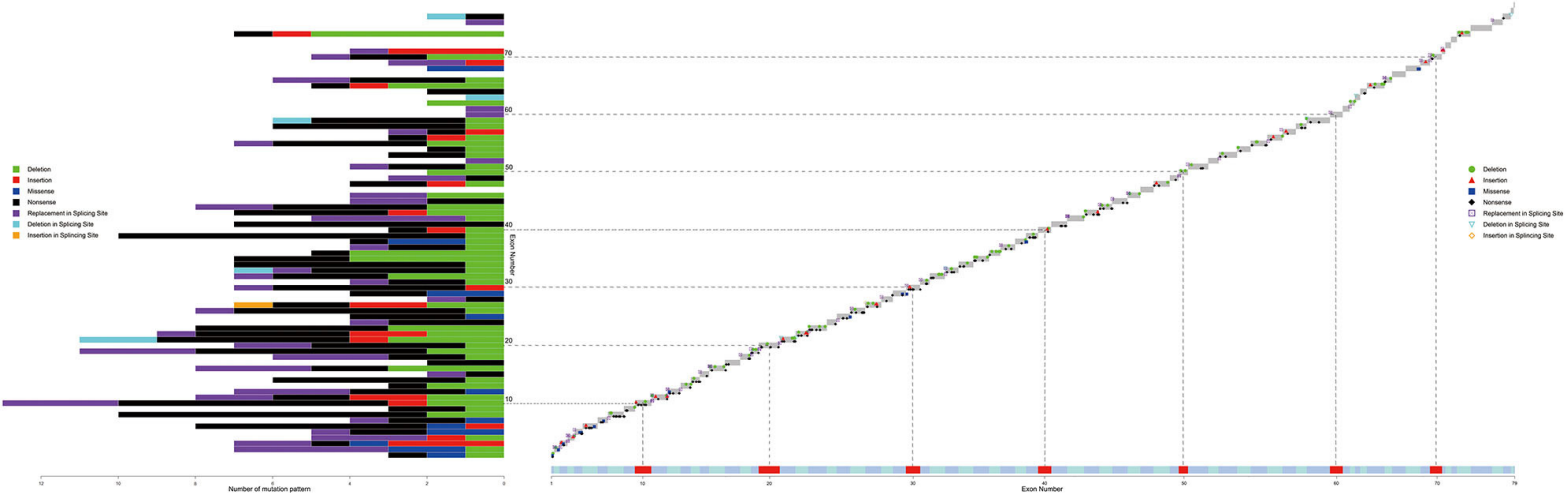
Distribution of different kind of SNVs in 79 exons. Different color represents with different kind of SNVs (deletion, insertion, missense, non-sense, replacement/deletion/insertion in splicing sites). Dash lines and blocks of colors were used as markers of every 10 exons. SNV, single or oligo nucleotide variations.

As shown in Figure 4, SNVs (non-sense, missense, small deletions and insertions, splice site mutations) were found among 72/79 exons. Exon 10 was accompanied by the highest number of SNVs (13 SNVs), and no hotspot existed. Chi-square tests, along with Cullen and Frey graphs, indicated that SNVs were distributed uniformly among all exons (Supplementary Figure 2).

Splicing site mutations were classified into three types: deletion, insertion, and replacement at the splice site, as shown in Figure 4. Five patients with DMD had a splice-site deletion, one patient with BMD had a splice-site insertion, and 42, 19, and, 4 patients with DMD, BMD, and IMD, respectively, had a splice-site replication.

### Ultrarare Mutations

Table 2 presents the details of ultrarare patients in our database. A total of 10 patients were identified to have rare mutations. First, there were four patients diagnosed with deep intronic mutations. Among them, two patients were had definite causative mutation while the other two patients only had defects at mRNA level. Four patients had large deletions across the adjacent intron and exon, which illustrated that the breakpoints could also be located in exons. Another two distinctive patients were identified. (1) A BMD patient with a large triplication. To our knowledge, this was the first reported triplication mutation on *DMD* gene. (2) A patient with BMD had a chimeric non-sense mutation. In the blood sample, the ratio of non-sense mutation to the wild type was about 8:2.

**Table 2:** Patients with ultra-rare mutations.

**Patient number**	**Disease type**	**Mutation**	**Specific characteristic**
1	BMD	c.31 + 36947G>A (intron1)	Deep intronic mutation
2	BMD	intron21 insertion 88bp (mRNA resequencing)	Deep intronic mutation
3	DMD	intron48 insertion 161bp (3 parts) mRNA resequencing	Deep intronic mutation
4	DMD	c.8217 + 18052A>G (intron55)	Deep intronic mutation
5	BMD	c.7661–14027_7700delinsCTGTTGCCTCCGGTTCTG	Mutation across adjacent intron and exon, large deletion with small insertion.
6	DMD	c.2623–1641_c.2687del (intron20 + exon21)	Mutation across adjacent intron and exon
7	DMD	exon45_46dup (exon46 partial dup)	Mutation across adjacent intron and exon
8	DMD	exon45del (partial del)	Mutation across adjacent intron and exon
9	BMD	c.5867G>A (p.Trp1956Ter, exon41) chimera	Patient with mosaic mutation
10	BMD	exon45_55triplication	Triplication

Table 3 shows eleven patients with two separate mutations. Both mutation patterns in the first three patients were observed more than once in the database, while in the other eight patients, at least one mutation pattern was unique. After further tests to see if the two mutations occurred independently, the results of nine patients did not support that the two mutations were independent (*p* < 0.05), while the results of the other two patients did not allow the rejection of the hypothesis (*p* = 0.077 and *p* = 0.260).

**Table 3:** Patients with two separate mutations.

**Patient number**	**Disease type**	**Mutation type**	**Mutation number in database**	Mutation frequency in database^*^	***P*-value^**#**^**
1	DMD	exon8del	2	0.12%	**0.033**
		exon52del	28	1.66%	
2	DMD	exon45_47del	67	3.98%	0.077
		exon51_52dup	2	0.12%	
3	DMD	exon48del	18	1.07%	0.260
		exon52del	28	1.66%	
4	DMD	exon45dup	1	0.06%	** <0.001**
		exon48_63dup	1	0.06%	
5	DMD	exon54_59del	1	0.06%	** <0.001**
		exon61_75del	1	0.06%	
6	DMD	Dp427c_exon2del	2	0.12%	**0.001**
		exon26_42del	1	0.06%	
7	DMD	exon 45_48dup	1	0.06%	**0.001**
		exon56_61dup	2	0.12%	
8	DMD	exon2del	1	0.06%	**0.047**
		exon48_50del	80	4.75%	
9	DMD	exon2dup	19	1.13%	**0.011**
		exon45_49dup	1	0.06%	
10	DMD	c.1590_1591delGA (p.Glu531ThrfsX3, exon13)	1	0.06%	** <0.001**
		exon60del	1	0.06%	
11	IMD	Dp427cdup	1	0.06%	** <0.001**
		exon45_57dup	1	0.06%	

* *Mutation frequency in database was calculated by (number of specific mutation)/(total number of patients with single CNV mutation)*.

# *P-value was calculated through a hypothesis test process under the following assumptions*.

*The frequency observed of each mutation pattern in the database were assumed to equal to the probability of occurrence of corresponding mutation pattern*.

*Null hypothesis: The two separate mutations happened independently*.

*Set α = 0.05 as the threshold*.

*P < 0.05 were considered as statistically significant to reject the null hypothesis*.

## Discussion

This study aimed to augment reliable data with uniform standards, including clinical features, genetic results, and treatment information, of patients with dystrophinopathy in China. The NRSDS-BMD/DMD database could complement the TREAT-NMD database, especially for researchers who are interested in Chinese or Asian patients, as this is the largest hospital-based dystrophinopathy database not only in China but also in Asian countries (21–23, 29–32). This research could also provide a valuable reference for future research on diagnostics and novel therapies.

PUMCH is the National Rare Disease Center in China. Patients from all over the country are referred to PUMCH by local hospitals. Thus, compared with previous Chinese DMD databases recruiting patients only from a part of China (21–23), patients included in this research came from all over China, except for three regions. As an inference, as shown in Figure 1, the annular contour line (shades of colors) indicated that the distance from PUMCH seemed to be the only factor influencing the registration rates of patients from each province. Ethnic background is important for genetic diseases. However, during our study, we found that most patients were Han Chinese, and the ethnic background of patients was similar to that of the Chinese population; thus, we did not display the results in the manuscript.

A distinct peak for dystrophinopathy diagnosis in our study was at 3 years, which may partially result from health screenings, especially the AST/ALT tests performed when children are enrolled in kindergartens in China. These children were initially suspected of having hepatopathy but finally diagnosed with DMD/BMD (21). Based on our study, some significant findings, such as delayed motor development, poor athletic performance at an early age, and higher serum enzymes could help parents and primary care physicians to consider DMD. Since patients with DMD had significantly higher enzyme levels than those with BMD, we calculated the cutoff values of all six biochemical enzyme levels in DMD and BMD. Different serum enzyme levels can help primary care physicians to distinguish DMD and BMD preliminarily. The benefits of steroid treatment for prolonged function are confirmed, and its regular use in affected children leads to additional improvements (33). In our database, 5–14 year patient group had the highest ratio in steroid therapy, which is consistent with the practice guidelines.

In our study, we also observed different distribution patterns of CNVs and SNVs. An apparent hotspot region located in exons 45–54 was observed in deletions, but duplications were distributed evenly along the whole gene, while they were previously reported to cluster in proximal or distal exons (19, 34). Our results suggested that different mechanisms might underlie the occurrence of deletions and duplications, yet in previous literature, similar recombination mechanisms, such as non-allelic homologous recombination (NAHR) and non-homologous end joining (NHEJ), were reported in the formation of deletions and duplications (35–41). More analyses, targeted at the genomic architecture near the breakpoints of patients with deletions and duplications, are needed to explore specific mechanisms. Moreover, intron 44 harbored more than a quarter (26.5%) of the starting points of deletions in our study. Intron 44 is the largest in *DMD*, accounting for 12% of its length, which was previously reported as a major breakpoint start (42, 43). The percentage is far more out of proportion to its length, which may not only result from the large size but also the specific genomic architecture. However, only a few studies have explored the potential mechanisms for intron 44 functioning as a major deletion breakpoint start. Blonden et al. (44) mapped the breakpoints in the distal region of intron 44 of 113 patients and found no significant frequent sequence. Miyazaki et al. (45) sequenced deletion breakpoints in three patients with DMD and found no association with particular sequence elements. However, Miyazaki et al. (45) discovered several palindromic sequences and short tandem repeats adjacent to the breakpoints. However, the two studies were conducted more than 10 years ago; therefore, more analyses based on new sequencing technologies are warranted.

SNVs are usually caused by unstable DNA polymerase activity associated with DNA replication or repair mistakes (46). In our study, Chi-square tests along with Cullen and Frey graph showed that SNVs were distributed uniformly among all exons (Supplementary Figure 1), which was reported in the previous literature (32), suggesting that SNVs in *DMD* are generated by chance. Many SNVs after exon 71 were predicted to cause premature termination, which in turn resulted in the DMD phenotype. However, experiments conducted on animal models showed different results. A transgenic mouse with truncated dystrophin, bearing a deletion of exon 71–78, displayed nearly normal muscle functions and normal DAP complex (47). Crawford also reported that syntrophin and dystrobrevin localize in the muscle membrane without the COOH-terminal domain of dystrophin (47). This region has a high frequency in alternative-splicing. In our database, most of the patients suffering from out-of-frame SNVs after exon 71 only developed the milder BMD phenotype, which also illustrated that the domains after exon 71 played a less critical function. Therefore, exons 71–79 can be elided when constructing the mini or micro-dystrophin loaded by adeno-associated virus (AAV) in promising gene transfer therapy.

Some rare mutation types of dystrophinopathy were also found in our database. Until now, there has been a lack of analyses targeted at deep intronic sequences and functions in patients with dystrophinopathy. We found that four patients in our database were diagnosed with a deep intronic mutation. Bovolenta reported two patients with intronic mutations, associated with abnormal splicing of *DMD* mRNA (48). Mutations in deep introns are related to diseases via the regulation of pre-mRNA splicing (49, 50). Large deletion breakpoints located in the exonic region showed the possibility of double-strand breaks and misregulation of DNA repair in the coding domain. Other rare mutation types in our database included somatic mosaicism and triplication in two BMD patients, which has only been reported in a few case reports (51, 52).

There were 11 (0.5%) patients with DMD in our database, who suffered from two separate mutations simultaneously. Most of these patients (No. 4–11) suffered from at least one mutation that was not observed in other patients in the database. In addition, the hypothesis test showed that some mutations (patient No. 1 and 4–11) did not occur independently, indicating that two or more rare mutations might coincide via related mechanisms. However, according to the test hypothesis results, we could not deny that relatively common mutations (mutations with higher frequency in the whole database) in patients 2 and 3 occurred independently. We supposed that there were different mechanisms underlying two mutations in the same patient, and we are looking forward to any possible experiments on this point in the future.

Non-sense read-through and exon skipping therapies are two promising genetic therapies under rapid development. In our database, 10.4% of DMD patients would benefit from potential non-sense read-through therapy. Deletion of exons 45–55, established using exon skipping therapy transformed DMD phenotypes to a milder BMD phenotype (53), from which 49.4% of DMD patients (72.1% of all DMD deletions) in our database would benefit. Currently, a clinical trial for ataluren (PTC124) is ongoing in a few hospitals in China (No. CTR20180881). In the future, our database will provide essential information for more trials about promising therapeutic and personalized therapies for Chinese patients with DMD.

There are some limitations to this study, which warrant further investigation. First, this is a single-center database, which might limit the generalizability of the study results. However, patients included in this study were from nearly all provinces in China, which contributed to the heterogeneity of the study population. Second, intron sequencing was not conducted due to cost and time. More studies should be performed to determine if certain DNA structures in intron 44 are related to large deletion initiations. Third, detailed information about the present steroid therapy or cardiac and pulmonary management is not included in this article. Data on clinical features were incomplete for some patients. We have been keeping monitoring patients' cardiac complications. ECG and UCG were conducted once a year among patients aged from 7 to 10 years old and twice a year after. Proper treatment would be taken if the dilated cardiomyopathy was developed. The follow-up results were not presented in this manuscript. In the future, we will continue to recruit more patients and perform long-term follow-ups. Finally, genetic data of parental carriers were not included in the database due to privacy reasons. We will continuously collect complete familial data as much as possible, especially for patients with ultrarare mutations in future follow-ups under their consent. We are also willing to cooperate with other medical centers in China to construct a multi-center DMD/BMD database. Novel genetic therapies, including exon skipping therapy and gene transfer using AAV, are currently being developed. Our database will provide comprehensive information to improve these therapies and design clinical trials, thus benefiting patients with DMD/BMD in China and other countries.

## Conclusions

We established a comprehensive Chinese dystrophinopathy database based on the hospital registry. Demographic data, biomarkers, clinical features, mutation characteristics, and therapeutic interventions were collected and systemically analyzed. The database serves as a valuable tool in diagnostics, scientific research, and clinical trials.

## Data Availability Statement

The datasets generated for this study are available on reasonable request to the corresponding author.

## Ethics Statement

The studies involving human participants were reviewed and approved by Ethics committee of the Peking Union Medical College Hospital. Written informed consent to participate in this study was provided by the participants' legal guardian/next of kin.

## Author Contributions

Y-RT and YD were responsible for conception and design of the study. Y-RT and CG were responsible for analyzing data and drafting manuscript. Y-ZG, Y-HZ, H-TR, F-XY, CL, D-CW, and L-YC were responsible for data collection. S-YZ and YD were responsible for revising the manuscript and figures. All authors contributed to the article and approved the submitted version.

## Conflict of Interest

The authors declare that the research was conducted in the absence of any commercial or financial relationships that could be construed as a potential conflict of interest.

## Supplementary Material

Supplementary Table 1 Registered patients and registration rate in each province.

Supplementary Table 2 Biomarkers and corresponding ROC.

Supplementary Table 3 Treatment characteristics and clinical features in different age groups.

Supplementary Figure 1 Flowchart of patients inclusion into the NRDRS-DMD/BMD database.

Supplementary Figure 2 Cullen and Frey graph of distribution of SNVs.

## Abbreviations

DMD: Duchenne muscular dystrophy
BMD: Becker muscular dystrophy
CNVs: copy number variations
SNVs: single or oligo nucleotide variations
CK: creatine kinase
LDH: lactate dehydrogenase
PUMCH: Peking Union Medical College Hospital
NRDRS: National Rare Disease Research System of China
IMD: intermediate muscular dystrophy
ROC: receiver operating characteristic curves
CK-MB: creatine kinase-muscle/brain
HBD: hydroxybutyric dehydrogenase
AST: aspartate aminotransferase
ALT: alanine aminotransferase
AUC: area under the curve
AAV: adeno-associated virus
MLPA: multiplex ligation-dependent probe amplification
NGS: next-generation sequencing
NAHR: non-allelic homologous recombination
NHEJ: non-homologous end joining.
