# Supplementary material for: A Comprehensive Analysis of 2013 Dystrophinopathies in China: A Report From National Rare Disease Center
Source: Front Neurol. 2020 Sep 30;11:572006. doi: 10.3389/fneur.2020.572006 (PMC7554367; doi:10.3389/fneur.2020.572006)
Supplement: Supplementary Table 1 — Registered patients and registration rate in each province. [file Table_1.DOCX]

Supplementary table 1. Registered patients and registration rate in each province

| Province | Number of registered patients | Registration rate(1 in million) |
| --- | --- | --- |
| ANHUI | 86 | 1.35 |
| BEIJING | 120 | 5.57 |
| FUJIAN | 28 | 0.7 |
| GUIZHOU | 20 | 0.56 |
| GANSU | 29 | 1.1 |
| GUANGDONG | 27 | 0.23 |
| GUANGXI | 7 | 0.14 |
| HAINAN | 4 | 0.42 |
| HEBEI | 280 | 3.7 |
| HENAN | 255 | 2.33 |
| HEILONGJIANG | 47 | 1.25 |
| HUBEI | 55 | 0.93 |
| HUNAN | 56 | 0.81 |
| JILIN | 32 | 1.18 |
| JIANGSU | 98 | 1.21 |
| JIANGXI | 37 | 0.79 |
| LIAONING | 76 | 1.75 |
| INNER MONGOLIA | 54 | 2.13 |
| NINGXIA | 19 | 2.73 |
| QINGHAI | 1 | 0.16 |
| SHANDONG | 247 | 2.45 |
| SHANXI(PROCINCIAL CAPITAL: TAIYUAN) | 80 | 2.15 |
| SHANXI(PROCINCIAL CAPITAL: XIAN) | 90 | 2.32 |
| SHANGHAI | 15 | 0.62 |
| SICHUAN | 48 | 0.58 |
| TIANJIN | 36 | 2.3 |
| XINJIANG | 25 | 1.01 |
| YUNNAN | 19 | 0.39 |
| ZHEJIANG | 64 | 1.09 |
| CHONGQING | 23 | 0.74 |
| HONG KONG | 1 | 0.13 |
| TIBET | 0 | / |
| MACAU | 0 | / |
| TAIWAN | 0 | / |

*34 patients in the database were lack of region data
