# Supplementary material for: A Comprehensive Analysis of 2013 Dystrophinopathies in China: A Report From National Rare Disease Center
Source: Front Neurol. 2020 Sep 30;11:572006. doi: 10.3389/fneur.2020.572006 (PMC7554367; doi:10.3389/fneur.2020.572006)
Supplement: Supplementary Table 2 — Biomarkers and corresponding ROC. [file Table_2.DOCX]

|  | **Cut-off value** | **Specificity** | **Sensitivity** | **AUC** |
| --- | --- | --- | --- | --- |
| **CK** | 8417 | 0.889 | 0.827 | 0.922 |
| **CK-MB** | 147.5 | 0.959 | 0.808 | 0.943 |
| **LDH** | 813 | 0.904 | 0.817 | 0.936 |
| **AST** | 175 | 0.839 | 0.831 | 0.904 |
| **ALT** | 218.5 | 0.864 | 0.848 | 0.92 |
| **HBD** | 612.5 | 0.892 | 0.77 | 0.898 |

Supplementary Table 2. Biomarkers and corresponding ROC
