# Supplementary material for: A Comprehensive Analysis of 2013 Dystrophinopathies in China: A Report From National Rare Disease Center
Source: Front Neurol. 2020 Sep 30;11:572006. doi: 10.3389/fneur.2020.572006 (PMC7554367; doi:10.3389/fneur.2020.572006)
Supplement: Supplementary Table 3 — Treatment characteristics and clinical features in different age groups. [file Table_3.DOCX]

Supplementary Table 3. Treatment characteristics and clinical features in different age groups

| **Characteristics** | | **Age groups** | | | |
| --- | --- | --- | --- | --- | --- |
|  |  | **< 5** | **5-15** | **16-24** | **≥25** |
| **Clinical features in DMD patients** | | | | | |
| Family history, n (%) | No | 150(67.9%) | 842(68.9%) | 26(28.6%) | 1(12.5%) |
|  | Yes | 17(7.7%) | 105(8.6%) | 8(8.8%) | 1(12.5%) |
|  | Unknown | 54(24.4%) | 275(22.5%) | 56(62.6%) | 6 (75.0%) |
| Ability of crawling, n (%) | No | 67(30.3%) | 535(43.8%) | 14 (15.4%) | 0(0.0%) |
|  | Poor | 79(35.7%) | 135(11.0%) | 0(0.0%) | 0(0.0%) |
|  | Regular | 7(3.2%) | 8(0.7%) | 0(0.0%) | 0(0.0%) |
|  | Unknown | 68(30.8%) | 544(44.5%) | 76(84.6%) | 8(100.0%) |
| Ability of running, n (%) | No | 7(3.2%) | 20(1.6%) | 0(0.0%) | 0(0.0%) |
|  | Slow | 2(0.9%) | 3(0.2%) | 34(37.4%) | 0(0.0%) |
|  | Regular | 134 (60.6%) | 873(71.4%) | 0(0.0%) | 1(12.5%) |
|  | Unknown | 78(35.3%) | 326(26.7%) | 56(62.6%) | 7(87.5%) |
| Gowers' sign, n (%) | + | 33(14.9%) | 342(28.0%) | 3(3.3%) | 0(0.0%) |
|  | ± | 39(17.6%) | 131(10.7%) | 2(2.2%) | 0(0.0%) |
|  | - | 3(1.4%) | 109(8.9%) | 1(1.1%) | 0(0.0%) |
|  | Unknown | 146(66.1%) | 640(52.4%) | 85(94.5%) | 1(100.0%) |
| Calf pseudohypertrophy, n (%) | + | 133(60.2%) | 867(70.9%) | 31(34.1%) | 0(0.0%) |
|  | ± | 18(8.1%) | 86(7.0%) | 2(2.2%) | 1(87.5%) |
|  | - | 2(0.9%) | 8(0.7%) | 1(1.1%) | 0(0.0%) |
|  | Unknown | 68(30.8%) | 261(21.4%) | 56(62.6%) | 0(0.0%) |
| Forearm pseudohypertrophy, n (%) | + | 60(27.1%) | 511(41.8%) | 17(18.7%) | 0(0.0%) |
|  | ± | 46(20.8%) | 246(20.1%) | 5(5.5%) | 0(0.0%) |
|  | - | 30(13.6%) | 185(15.1%) | 2(2.2%) | 0(0.0%) |
|  | Unknown | 85(38.5%) | 280(22.9%) | 66(73.6%) | 8(100.0%) |
| **Clinical features in BMD patients** | | | | | |
| Family history, n (%) | No | 34(51.5%) | 97(49.7%) | 22(41.5%) | 17(31.5%） |
|  | Yes | 2(3.0%) | 37(19.0%) | 8(15.1%) | 15(27.8%) |
|  | Unknown | 30(45.5%) | 61(31.3%) | 23(43.4%) | 22(40.7%) |
| Ability of crawling, n (%) | No | 7(10.6%) | 41(21.1%) | 1(1.9%) | 3(5.6%) |
|  | Poor | 15(22.7%) | 18(9.2%) | 2(3.8%) | 1(1.8%) |
|  | Regular | 10(15.2%) | 16(8.2%) | 1(1.9%) | 3(5.6%) |
|  | Unknown | 34(51.5%) | 120(61.5%) | 49(92.4%) | 47(87.0%) |
| Ability of running, n (%) | No | 0(0.0%) | 0(0.0%) | 0(0.0%) | 0(0.0%) |
|  | Slow | 19(28.8%) | 52(26.7%) | 13(24.5%) | 9(16.7%) |
|  | Regular | 9(13.6%) | 27(13.8%) | 1(1.9%) | 2(3.7%) |
|  | Unknown | 38(57.6%) | 116(59.5%) | 39(73.6%) | 43(79.6%) |
| Gowers' sign, n (%) | + | 2(3.0%) | 3(1.5%) | 8(15.1%) | 14(25.9%) |
|  | ± | 0(0.0%) | 7(3.6%) | 2(3.8%) | 5(9.3%) |
|  | - | 15(22.8%) | 99(50.8%) | 13(24.5%) | 1(1.9%) |
|  | Unknown | 49(74.2%) | 86(44.1%) | 30(56.6%) | 34(62.9%) |
| Calf pseudohypertrophy, n (%) | + | 31(47.0%) | 110（56.4%） | 31(58.5%) | 30(55.6%) |
|  | ± | 4(6.0%) | 27（13.8%） | 0(0.0%) | 1(1.9%) |
|  | - | 0(0.0%) | 5(2.6%) | 0(0.0%) | 0(0.0%) |
|  | Unknown | 31(47.0%) | 53(27.2%) | 22(41.5%) | 23(42.5%) |
| Forearm pseudohypertrophy, n (%) | + | 11(16.7%) | 64(32.8%) | 25(47.2%) | 25(46.2%) |
|  | ± | 11(16.7%) | 38(19.6%) | 5(9.4%) | 3(5.6%) |
|  | - | 11(16.7%) | 35(17.9%) | 0(0.0%) | 1(1.9%) |
|  | Unknown | 33(49.9%) | 58(29.7%) | 23(43.4%) | 25(46.3%) |
| **Clinical features in IMD patients** | | | | | |
| Family history, n (%) | No | 13(72.2%) | 46(67.6%) | 2(20.0%) | 1(20.0%) |
|  | Yes | 0(0.0%) | 10(14.7%) | 4(40.0%) | 1(20.0%) |
|  | Unknown | 5(27.8%) | 12(17.6%) | 4(40.0%) | 3(60.0%) |
| Ability of crawling, n (%) | No | 2(11.1%) | 23(33.8%) | 3(30.0%) | 1(20.0%) |
|  | Poor | 9(50.0%) | 16(23.5%) | 0(0.0%) | 1(20.0%) |
|  | Regular | 2(11.1%) | 4(5.9%) | 0(0.0%) | 0(0.0%) |
|  | Unknown | 5(27.8%) | 25(36.8%) | 7(70.0%) | 3(60.0%) |
| Ability of running, n (%) | No | 1(5.6%) | 1(1.5%) | 0(0.0%) | 0(0.0%) |
|  | Slow | 12(66.7%) | 52(76.5%) | 2(20.0%) | 2(40.0%) |
|  | Regular | 0(0.0%) | 2(2.9%) | 0(0.0%) | 0(0.0%) |
|  | Unknown | 5(27.8%) | 13(19.1%) | 8(80.0%) | 3(60.0%) |
| Gowers' sign, n (%) | + | 0(0.0%) | 24(35.3%) | 2(20.0%) | 0(0.0%) |
|  | ± | 2(11.1%) | 11(16.2%) | 0(0.0%) | 0(0.0%) |
|  | - | 2(11.1%) | 5(7.4%) | 0(0.0%) | 0(0.0%) |
|  | Unknown | 14(77.8%) | 28(41.2%) | 8(80.0%) | 5(100.0%) |
| Calf pseudohypertrophy, n (%) | + | 12(66.7%) | 51(75.0%) | 5(50.0%) | 2(40.0%) |
|  | ± | 0(0.0%) | 6(8.8%) | 0(0.0%) | 0(0.0%) |
|  | - | 0(0.0%) | 1(1.5%) | 0(0.0%) | 0(0.0%) |
|  | Unknown | 6(33.3%) | 10(14.7%) | 5(50.0%) | 3(60.0%) |
| Forearm pseudohypertrophy, n (%) | + | 4(22.2%) | 32(47.1%) | 3(30.0%) | 0(0.0%) |
|  | ± | 4(22.2%) | 15(22.1%) | 2(20.0%) | 0(0.0%) |
|  | - | 2(11.1%) | 8(11.8%) | 0(0.0%) | 0(0.0%) |
|  | Unknown | 8(44.4%) | 13(19.1%) | 5(50.0%) | 5(50.0%) |
| **Treatment in DMD patients** | | | | | |
| Steroid Use | Currently receiving Steroids | 141(63.8%) | 450(36.8%) | 1(1.1%) | 0(0.0%) |
|  | Previously receiving steroids | 3(1.4%) | 539(44.1%) | 37(40.7%) | 4(50.0%) |
|  | Never receiving steroids | 60(27.1%) | 98(8.0%) | 12(13.2%) | 4(50.0%) |
|  | Unknown | 17(7.7%) | 135(11.1%) | 40(45.1%) | 0(0.0%) |
| Ventilation | Not ventilated | 216(97.7%) | 1083(88.6%) | 42(46.2%) | 1(12.5%) |
|  | Part-time non-invasive ventilation | 0(0.0%) | 3(0.2%) | 6(6.6%) | 1(12.5%) |
|  | Full-time ventilation | 0(0.0%) | 0(0.0%) | 2(2.2%) | 1(12.5%) |
|  | Other ventilation | 0(0.0%) | 0(0.0%) | 1(1.1%) | 1(12.5%) |
|  | Unknown | 5(2.3%) | 136(11.1%) | 40(45.0%) | 4(50.0%) |

*4 DMD patients were excluded due to the missing of accurate age.
