# Supplementary figures and images for: A Comprehensive Analysis of 2013 Dystrophinopathies in China: A Report From National Rare Disease Center
Source: Front Neurol. 2020 Sep 30;11:572006. doi: 10.3389/fneur.2020.572006 (PMC7554367; doi:10.3389/fneur.2020.572006)

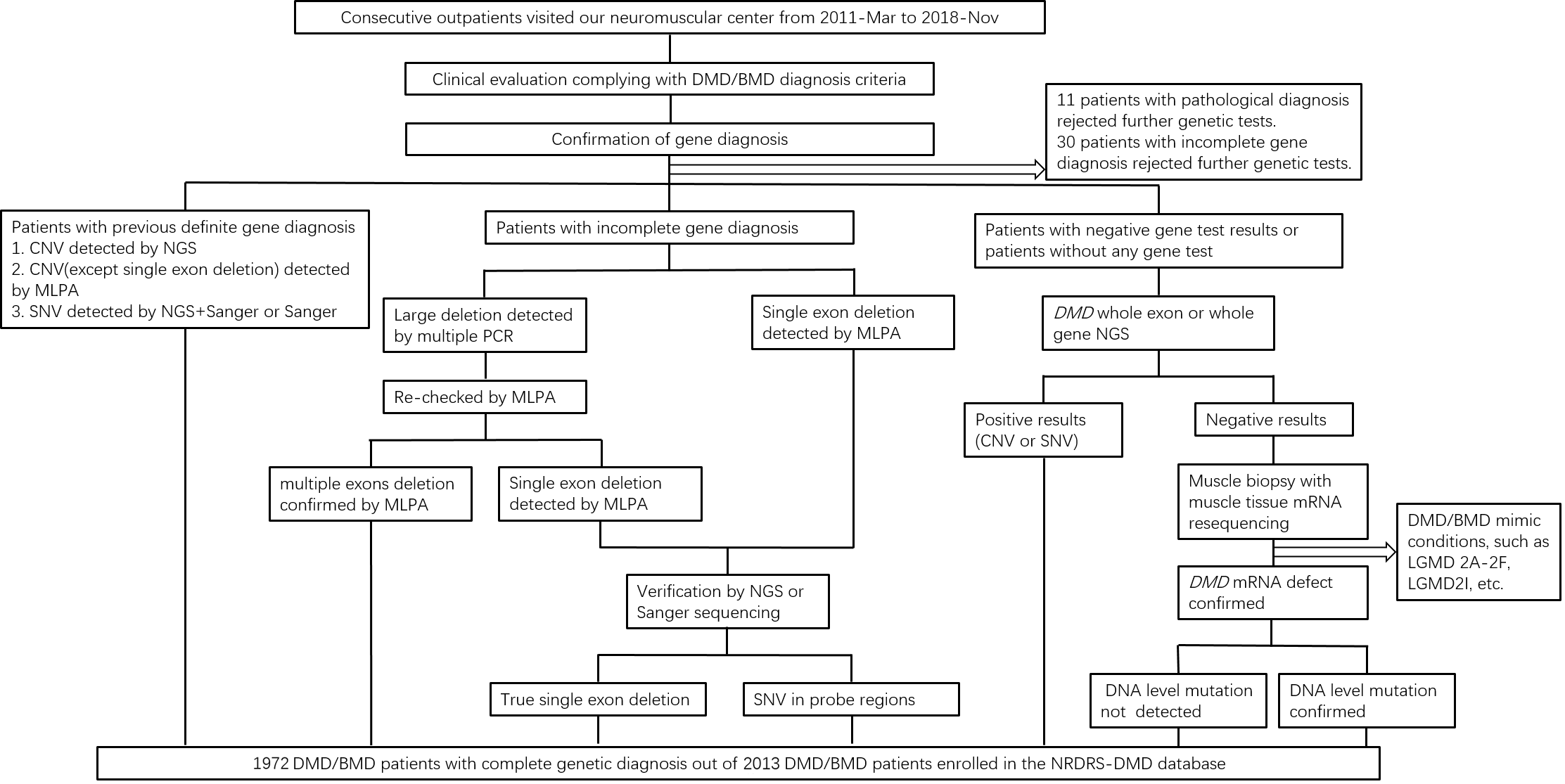

Supplement: Supplementary Figure 1 — Flowchart of patients inclusion into the NRDRS-DMD/BMD database. [file Image_1.tif]

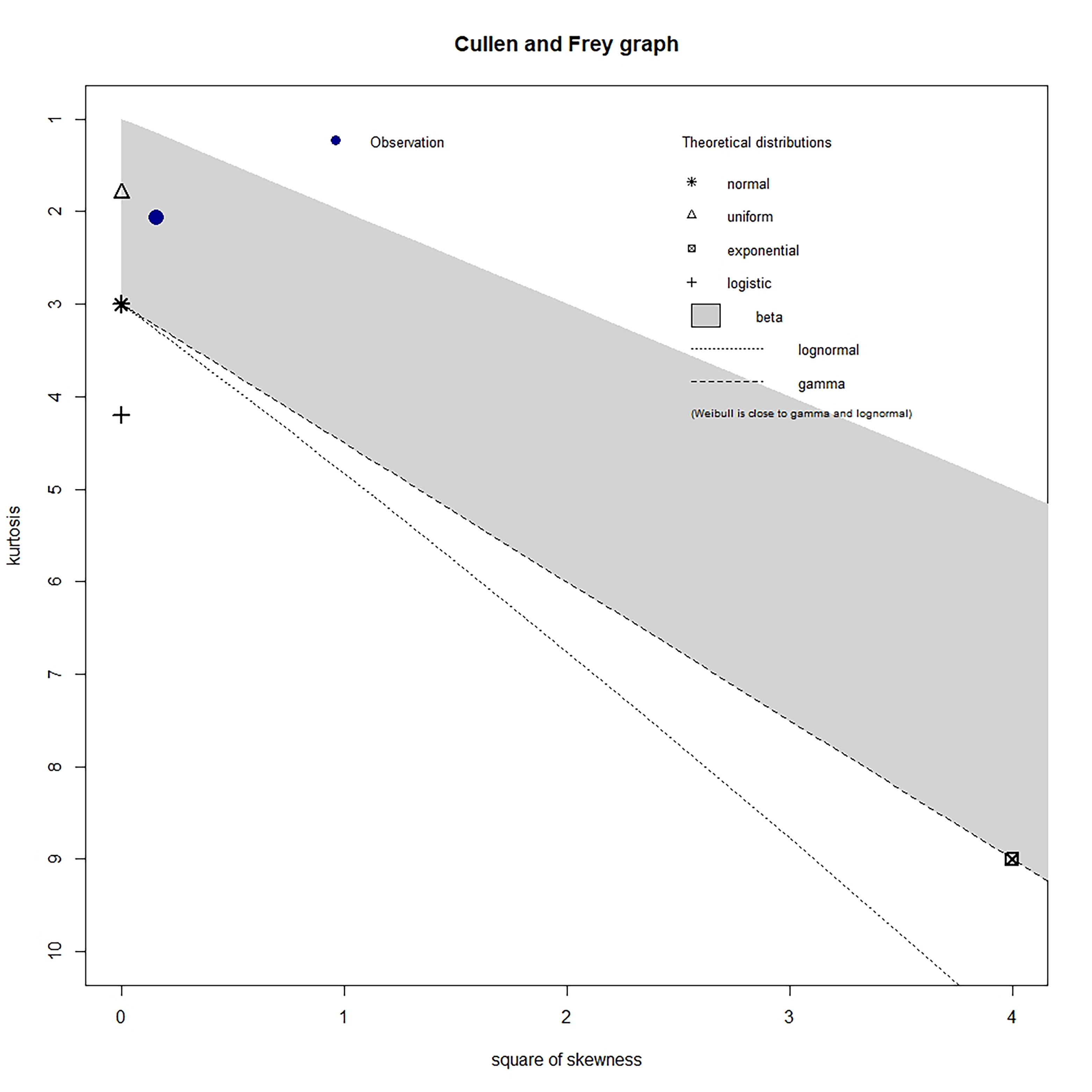

Supplement: Supplementary Figure 2 — Cullen and Frey graph of distribution of SNVs. [file Image_2.TIF]
